# Multicohort transcriptome analysis of whole blood identifies robust human response signatures in *Plasmodium falciparum* infections

**DOI:** 10.1186/s12936-022-04374-5

**Published:** 2022-11-15

**Authors:** Yan-hui Zhang, Xin-zhuan Su, Jian Li, Jia-jian Shi, Li-hua Xie

**Affiliations:** 1 Key Laboratory of Gastrointestinal Cancer (Fujian Medical University), Ministry of Education, Fuzhou, China; 2 Laboratory of Malaria and Vector Research, National Institute of Allergy and Infectious Disease, National Institutes of Health, Bethesda, MD USA; 3 State Key Laboratory of Cellular Stress Biology, Innovation Center for Cell Signaling Network, School of Life Sciences, Xiamen University, Xiamen, Fujian China

**Keywords:** *Plasmodium falciparum*, Multicohort transcriptome analysis, Neutrophil

## Abstract

**Background:**

To understand how *Plasmodium falciparum* malaria is controlled, it is essential to elucidate the transcriptomic responses of the human host in naturally-exposed populations. Various individual studies of the human transcriptomic responses to naturally transmitted *P. falciparum* infections have been reported with varying results. Multicohort gene expression analysis by aggregating data from diverse populations into a single analysis will increase the reproducibility and reliability of the results.

**Methods:**

In this study, discovery cohorts GSE1124-GPL96, GSE34404, GSE117613, and validation cohort GSE35858 were obtained from the Gene Expression Omnibus. A meta-analysis using data from the multicohort studies was performed to identify the differentially expressed genes (DEGs) between malaria-infected and noninfected individuals using the MetaIntegrator R package. Subsequently, the protein–protein interaction (PPI) networks of the DEGs were constructed using Cytoscape software. Significant modules were selected, and the hub genes were identified using the CytoHubba and MCODE plug-ins. Multicohort WGCNA was conducted to find a correlation between modules and malaria infection. Furthermore, the immune cell profile of the peripheral blood in different groups was identified using ssGSEA.

**Results:**

These analyses reveal that neutrophil activation, neutrophil-mediated immunity, and neutrophil degranulation are involved in the human response to natural malaria infection. However, neutrophil cell enrichment and activation were not significantly different between mild malaria and severe malaria groups. Malaria infection also downregulates host genes in ribosome synthesis and protein translation and upregulates host cell division-related genes. Furthermore, immune cell profiling analysis shows that activated dendritic cells and type 2 T helper cells are upregulated, while activated B cells, immature B cells, and monocytes are downregulated in the malaria-infected patients relative to the noninfected individuals. Significantly higher enrichment of activated dendritic cell-related genes and significantly lower enrichment of monocyte-related genes are also observed in the peripheral blood of the severe malaria group than in the mild malaria group.

**Conclusion:**

These results reveal important molecular signatures of host responses to malaria infections, providing some bases for developing malaria control strategies and protective vaccines.

**Supplementary Information:**

The online version contains supplementary material available at 10.1186/s12936-022-04374-5.

## Background

Malaria remains one of the most serious infectious diseases worldwide. There were an estimated 241 million malaria cases and 627,000 malaria deaths worldwide in 2020, with sub-Saharan Africa bearing the highest proportions of the cases [1]. The majority of deaths are caused by infections involving *Plasmodium falciparum*. The lack of an effective malaria vaccine and the emergence of drug-resistant parasite strains are the major obstacles to the elimination of malaria. The severity of malaria ranges from asymptomatic to mild to severe complications such as severe anaemia and cerebral malaria. Understanding the impact of *P. falciparum* on the human host’s responses to parasite infections is crucial for designing disease control measures. A promising approach for investigating systemic host responses to malaria parasite infections is the transcriptional analysis of host genes using methods such as microarray and RNA sequencing analyses based on data collected from multiple independent cohort studies.

Several cohort studies using microarrays have investigated the human response to *Plasmodium falciparum* infections. Lee et al. reported differentially expressed genes (DEGs) associated with coma, hyperlactataemia, thrombocytopenia, and neutrophil granule–related genes between severe and uncomplicated malaria by RNA sequencing blood samples from infected patients [2]. Similarly, Rothen et al. identified DEGs linked to the regulation of transcription, cell cycle, phosphatidylinositol signaling, and erythrocyte development following controlled human malaria infection (CHMI) by performing RNA-Seq on the whole blood [3]. Additionally, Boldt et al. identified immunoglobulin production, complement regulation, and interferon-beta (IFN-b) signaling as the most discrepant features between uncomplicated malaria and other clinical presentations of *P. falciparum* infection using microarray analysis of the whole blood [4]. However, these individual cohort studies are often specific to specific human populations and may not reflect the heterogeneity observed in all the patient populations. Meta-analysis of data from multicohort studies integrating heterogeneous datasets may allow identifying robust and reproducible signatures by leveraging the biological and technical heterogeneity in the datasets.

In the present investigation, DEGs between malaria-infected and noninfected individuals from multicohort datasets were identified using the MetaIntegrator R package. A protein–protein interaction (PPI) network was constructed to study the association between the DEGs and to identify hub genes using different modules of Cytoscape software. Additionally, multicohort weighted gene coexpression network analysis [5] was performed by constructing the co-expression network to find correlations between gene modules and malaria infection. Furthermore, to investigate the variations in immune responses between different groups, the immune cell profile of peripheral blood was identified by calculating the scores of 28 immune signatures in each sample using single-sample gene set enrichment analysis (ssGSEA). These analyses reveal important information for a better understanding of the host-parasite interactions and for designing strategies to control malaria.

## Methods

### Data acquisition and DEG identification

The Gene Expression Omnibus (GEO) was searched for datasets on human gene responses to malaria with the following criteria: (I) Search term: {[*Plasmodium falciparum*] AND (human whole blood)} was used to retrieve datasets relating to the human transcriptomic response to *P. falciparum* infections; (II) each study should be conducted using whole blood, not peripheral blood mononuclear cells (PBMCs), as PBMCs contain few granulocytes after purification; and (III) the study should include uninfected controls. The datasets were identified and systematically downloaded from the GEO. The GEO accession number, platform, tissue, phenotype, and sample size were extracted from each identified dataset (Table 1). The differentially expressed genes of the cohorts were identified by an integrated multicohort analysis using the MetaIntegrator R package. Hedges’ g effect size was computed for each gene for each dataset. Cochrane’s Q value was calculated to evaluate the heterogeneity of effect size estimates between studies. Fisher's test was used for combining *P*-values across studies. Receiver operating characteristic (ROC) curves were used to demonstrate the classification performance of the Meta Score in both the discovery cohort and the validation cohort.

**Table 1:** whole blood transcriptional datasets of malaria infection from GEO

GEO ID	Platform	Tissue	Phenotype	Plasmodium species	Cases
GSE1124 [9]	GPL96	whole blood cells	asymptomatic, uncomplicated malaria, severe malaria anaemia, cerebral malaria, healthy children	*P. falciparum*	25
GSE117613 [10]	GPL10558	whole blood cells	cerebral malaria, severe malaria anaemia, community children without *Plasmodium falciparum* infection	*P. falciparum*	46
GSE34404 [11]	GPL10558	whole blood cells	symptomatic phase of blood-stage *Plasmodium falciparum* infection, age-matched controls	*P. falciparum*	155
GSE35858	GPL15240	whole blood cells	healthy, acute uncomplicated, and complicated *Plasmodium falciparum* malaria infection	*P. falciparum*	37

### Gene Ontology (GO) enrichment analysis and protein–protein interaction (PPI) network analysis

GO analysis of the DEGs, including biological process (BP), cellular component (CC), and molecular function (MF) enrichment analyses, was performed using the clusterProfiler package in the Bioconductor platform. pvalueCutoff = 0.05 and qvalueCutoff = 0.2 were designated as the thresholds for statistical significance and for determining significant enrichment. A bubble chart showing the *P*-value, fold enrichment, and gene counts were used to describe the essential GO terms. PPI information of the DEGs was acquired from the Search Tool for the Retrieval of Interacting Genes (STRING) database (http://www.stringdb.org/). Then, Cytoscape software v.3.8.1 was used to construct the PPI network. Hub genes (genes with high correlation in candidate modules) were identified using the CytoHubba plugin and the molecular complex detection (MCODE) plugin. The genes with high scores calculated by CytoHubba in the extracted network were considered to be hub genes that have high correlation or connectivity in candidate modules [6].

### Weighted correlation network analysis

Weighted correlation network analysis (WGCNA) is a systems biology method for finding co-expressed modules and hub genes [7]. Meta-analysis of data from two microarray studies can be performed using WGCNA to find repeatable, conserved, co-expressed gene modules [8]. The microarray datasets from similar platforms were preprocessed, and the comparability was assessed. Then, the data were subjected to WGCNA to identify functional modules. The highly conserved modules in the malaria-infected groups or control groups in the multicohort databases were identified separately, and functional analysis was subsequently performed using the clusterprofile R package.

### Single-sample gene set enrichment analysis (ssGSEA)

For each dataset, the enrichment of 28 immune signatures for each sample was quantified using ssGSEA, as implemented in the GSVA R package. The subsets of genes representative of specific immune cell types were identified from the ImmPort database (Additional file 10: Table S1). The ssGSEA scores (enrichment level) were compared between different groups in each dataset. Statistical analyses were performed using the unpaired *t*-test. All statistical analyses were performed using R software (version 4.0.2).

## Results

### Dataset description

Four studies analysing whole blood transcriptional responses to malaria infections were identified from the NCBI database GEO (Table 1). Each of these studies profiled the whole blood transcriptome of malaria-infected and control healthy individuals. Three of the cohorts were randomly chosen as discovery cohorts, and the remaining dataset, GSE35858, was used as a validation cohort.

### Integrated multicohort analysis of DEGs between malaria-infected and noninfected individuals

**Fig. 1:**
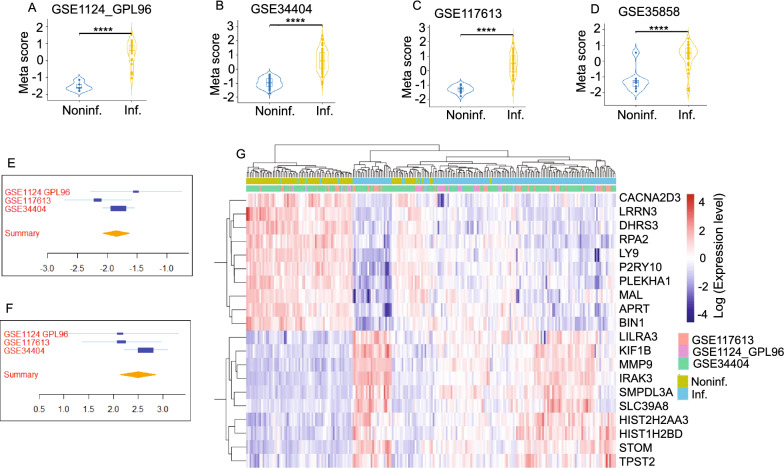
Differentially expressed gene (DEG) analysis between malaria-infected and noninfected groups by using the MetaIntegrator R package. **A**–**D**, Plots of Meta Scores from the four cohort datasets comparing data from malaria-infected and noninfected groups were. **E** and **F**, The forest plots of DGEs LRRN3 and MMP9 among the different discovery cohorts. A positive standardized mean difference indicates a higher level while a negative standardized mean difference indicates a lower level in the malaria-infected group relative to the noninfected group. **G**. Heatmap of top 10 upregulated genes and top 10 downregulated genes. The gene expression levels are normalized across samples, and red (upregulated) and blue(downregulated) are colour-coded according to the right colour bar. Colour keys on the top of the heatmap denote sample type and dataset

Using the MetaIntegrator R package, 887 significantly differentially regulated genes (349 upregulated and 538 downregulated) were identified with an FDR  ≤ 0.05 and an absolute effect size greater than or equal to 1 between malaria-infected and noninfected control individuals in the discovery datasets (Additional file 11: Table S2 and Additional file 12: Table S3). The area under the receiver operating characteristic (ROC) curve (AUC) values were measured to identify the ability of the DEGs to distinguish the malaria-infected group from the control group. In the discovery cohorts, the AUC values for the datasets of GSE1124-GPL96, GSE34404, and GSE117613 were 1.0, 0.98, and 0.99, respectively (Additional file 1: Fig. S1A). The ROC curve for the validation cohort GSE35858 was plotted, and the AUC value was 0.884, which was considered as having an excellent ability of the DEGs to distinguish the malaria-infected group from the control group (Additional file 1: Fig. S1B). The Meta Score which can be used to examine the identified DEGs was calculated for each sample based on the geometric mean of the upregulated genes minus the geometric mean of the downregulated genes. Significantly different Meta Scores were observed between the control group and the malaria-infected group in both the discovery cohorts and the validation cohort (Fig. 1A–D). The identified DEGs showed a consistent trend and statistical significance among the different discovery cohorts according to a forest plot. For example, LRRN3 was significantly down-regulated (Fig. 1E), whereas MMP9 was significantly upregulated in the infected groups (Fig. 1F). The heatmap of the top 10 upregulated genes and the top 10 downregulated genes are shown in Fig. 1G. Clear separation of gene expression patterns was observed in the noninfected and malaria-infected populations.

### Functional enrichment of DEGs and PPI network analysis

**Fig. 2:**
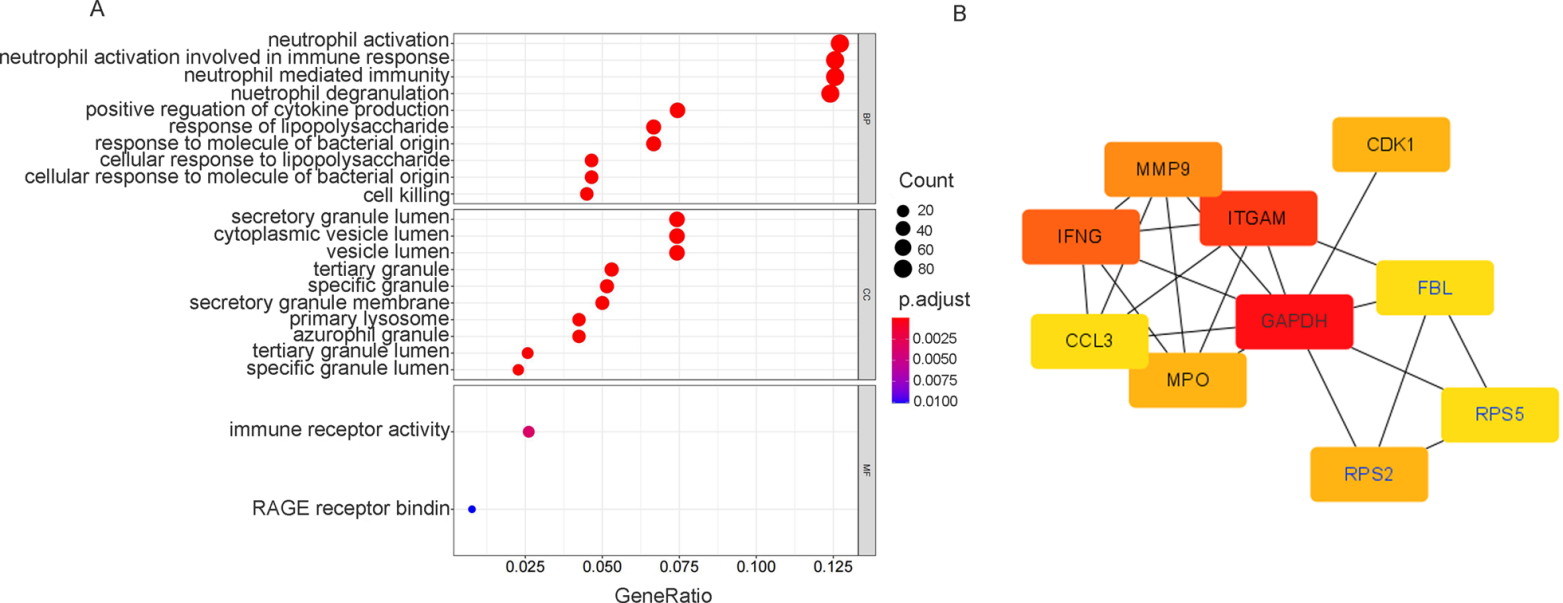
Functional enrichment of DEGs and hub gene identification. **A** GO enrichment analysis of 887 differentially expressed genes between the malaria-infected and noninfected groups in the discovery cohorts. ‘Gene ratio’ is the percentage of total DEGs in the given GO term. The size of the dots represents the number of genes in DEGs associated with the GO term and the colour of the dots represents the *P*-adjusted values. **B** The top 10 hub genes were identified based on the degree ranking method using the cytoHubba plug-in in Cytoscape. The rank of the connection degree is represented by different colours (from red to yellow). Genes in blue indicate downregulated genes, while genes in black indicate upregulated genes in the malaria-infected group

**Fig. 3:**
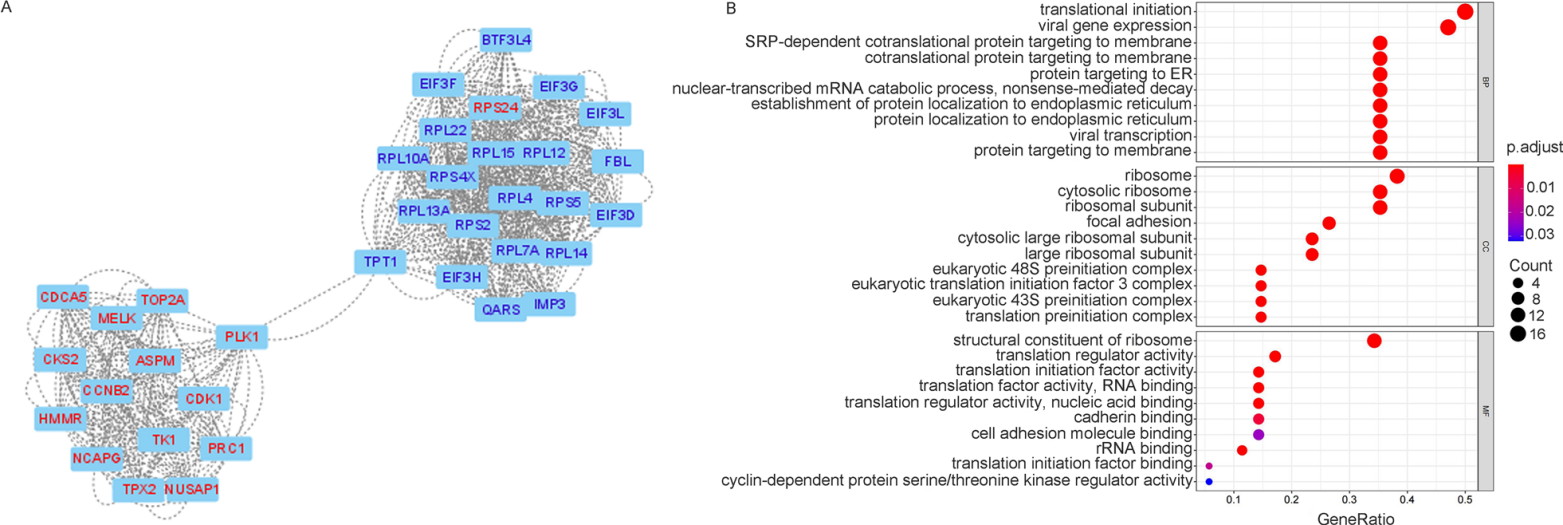
Module 1 of the DEGs was identified between malaria-infected and noninfected groups. **A** Module 1 of the DEGs was identified using the Molecular Complex Detection (MCODE) plug-in in Cytoscape. Genes in red are upregulated genes, while genes in blue are downregulated genes in the malaria-infected group. **B** GO enrichment analysis of M1 module of the DEGs. 'Gene ratio' is the percentage of total DEGs in the given GO term. The size of the dots represents the number of genes in DEGs associated with the GO term and the colour of the dots represents the *P*-adjusted values

**Fig. 4:**
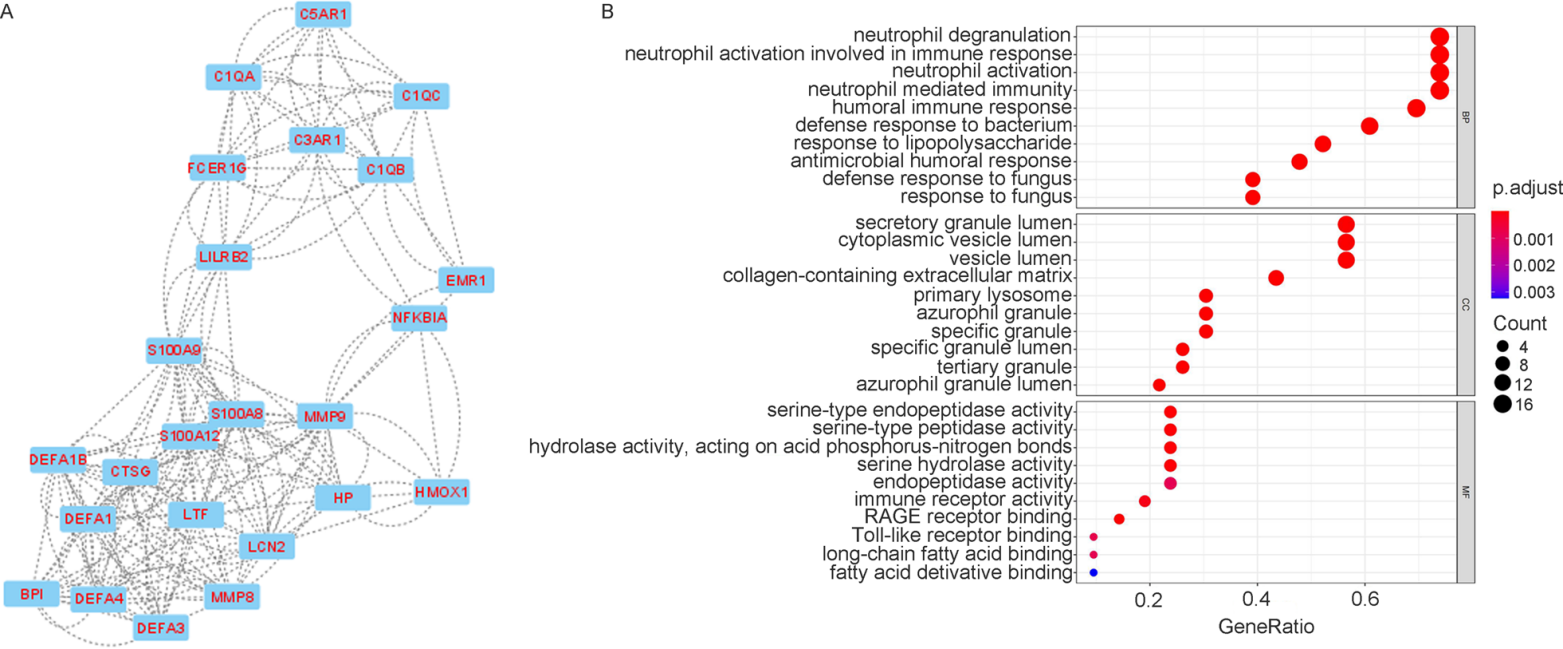
Module 3 of the DEGs identified between malaria-infected and noninfected groups. **A** M3 module of the DEGs was identified using MCODE plug-in in Cytoscape. Genes in red are upregulated genes in the malaria-infected group. **B** GO enrichment analysis of M3 module of the DEGs. ‘Gene ratio’ is the percentage of total DEGs in the given GO term. The size of the dots represents the number of genes in DEGs associated with the GO term and the colour of the dots represents the *P*-adjusted values

GO enrichment analysis was conducted on the 887 differentially expressed genes between the malaria-infected and noninfected groups using the clusterProfiler package. GO enrichment analysis showed that the highest enriched GO-terms in the infected groups were mainly related to neutrophil activation, neutrophil-mediated immunity, and neutrophil degranulation in the biological process (Fig. 2A). Associated with neutrophil functions, enrichment of GO-terms involved in the secretory granule lumen, vesicle lumen, tertiary granule, and specific granule in cellular components were also observed. Next, the DEGs were uploaded to STRING to obtain the PPI network. The result of STRING analysis was imported into Cytoscape, and the hub genes were identified based on the degree ranking method using the cytoHubba plug-in. The top ten hub genes were selected, consisting of seven upregulated genes and three downregulated genes (Fig. 2B). These genes could be considered as biomarkers for malaria infection. To identify functional units in the PPI network, module analysis was conducted using the Molecular Complex Detection (MCODE) plug-in. The top three modules were selected, with scores of 17.1 (M1), 9.4 (M2), and 8.5 (M3), respectively. M2 module contained 11 nodes, including SIN3A, NCOR2, KDM1A, HIST1H2BK, HIST1H3E, HIST1H2BD, KAT2A, DNMT1, HIST2H2BE, HIST2H2AA3, and HIST1H1C. These genes are mostly associated with chromatin organization (Additional file 2: Fig. S2). GO enrichment analysis was performed for M1 and M3, which had more genes. M1 was mainly involved in protein translation, protein localization, and ribosome synthesis (Fig. 3A, B). Further analysis showed that the upregulated genes in M1 were mainly associated with cell division (Additional file 3: Fig. S3A), while the downregulated genes were mainly involved in protein translation, localization, and ribosome synthesis (Additional file 3: Fig. S3B). M3 was mainly associated with neutrophil degranulation and activation, and related genes were upregulated in the malaria-infected group. (Fig. 4A, B). These results suggest that malaria infection can induce neutrophil degranulation, activation, and cell division but inhibit ribosome synthesis and protein translation, and localization.

### Identification of gene modules specific for malaria-infected and noninfected individuals

**Fig. 5:**
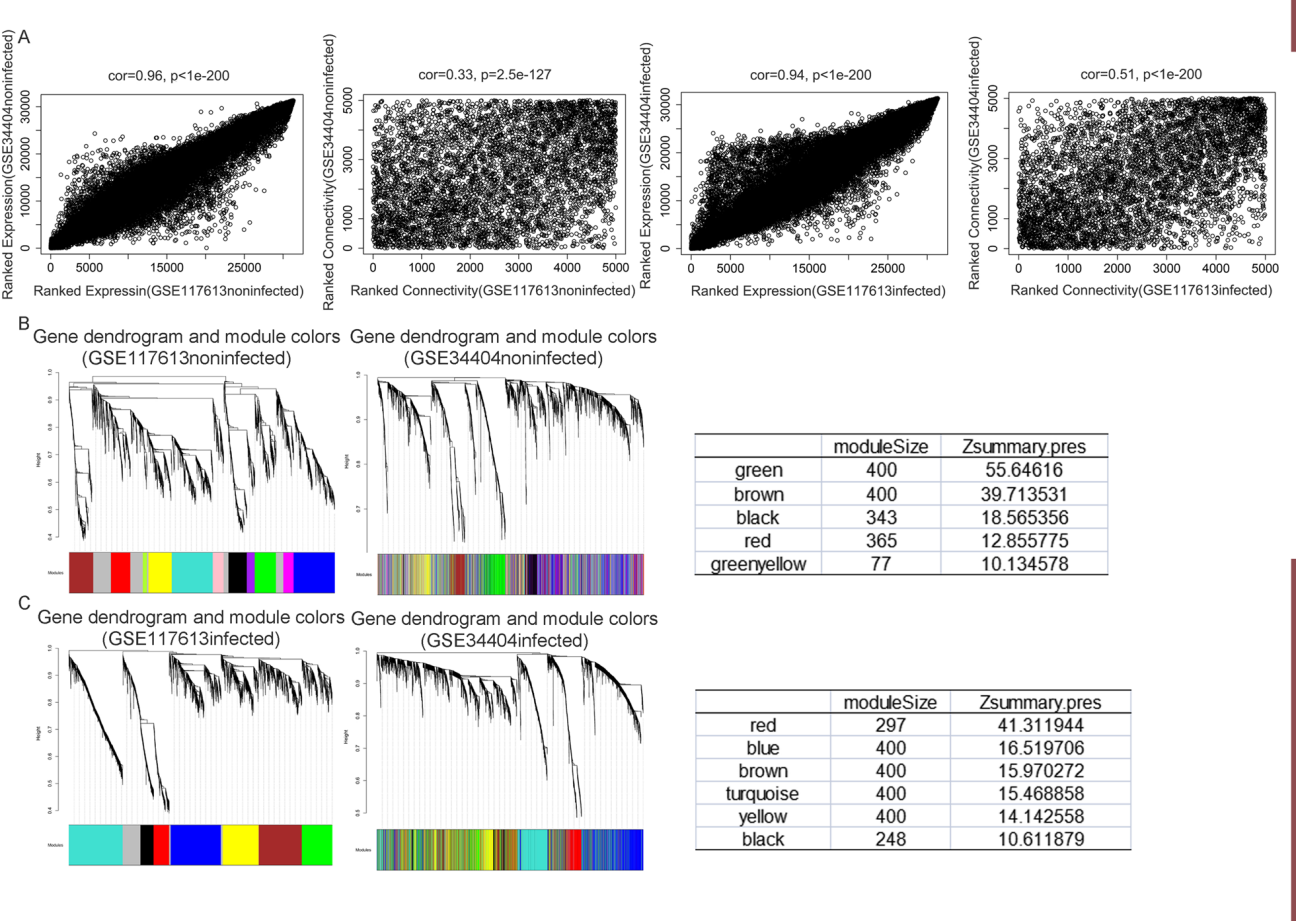
Conserved modules in noninfected and malaria-infected groups from the GSE117613 and GSE34404 datasets identified using multicohort WGCNA. **A** The correlations of average gene expression and overall connectivity in noninfected and malaria-infected groups between the GSE117613 and GSE34404. **B** Highly conserved modules in the noninfected group between the GSE117613 and GSE34404 datasets were identified. Modules that had a “Zsummary. Pres” value > 10 were considered highly conserved. **C** Highly conserved modules in the malaria-infected group between the GSE117613 and GSE34404 datasets were identified. Modules that had a “Zsummary.pres”" value  > 10 were considered highly conserved. The colours are used for labelling modules

**Fig. 6:**
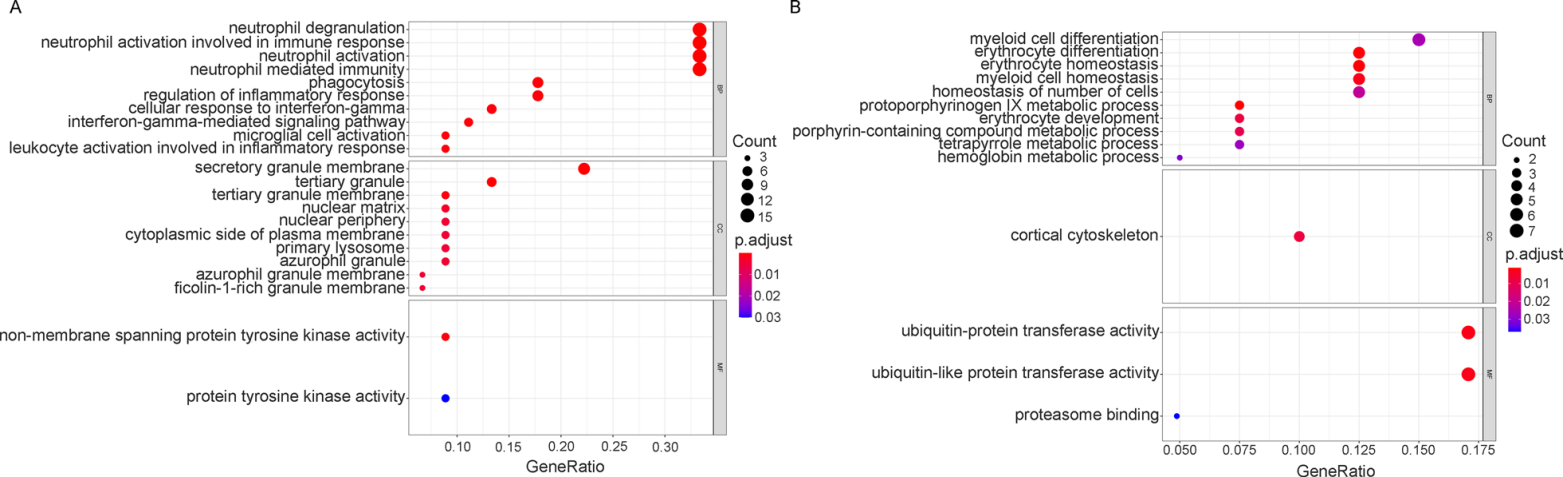
Different biological activities between the malaria-infected and noninfected groups identified using multicohort WGCNA. **A** GO enrichment analysis of the highly conserved module between GSE117613 and GSE34404 in the malaria-infected group that was not present in the normal noninfected group. **B** GO enrichment of the highly conserved module between GSE117613 and GSE34404 in the malaria-infected group that was not present in the noninfected group. ‘Gene ratio’ is the percentage of total DEGs in the given GO term. The size of the dots represents the number of genes in DEGs associated with the GO term and the colour of the dots represents the P-adjusted values

Using different analysis methods was necessary to confirm the above discoveries. WGCNA is a very useful method for studying gene co-expression, which is becoming increasingly common for identifying key modules associated with diseases. Multicohort WGCNA was performed to identify the conserved modules in malaria-infected groups or healthy controls between the GSE117613 and GSE34404 datasets, which used the same platform and comparable. GO function enrichment analysis was then used to assess the functions of genes in the conserved modules. The correlations of average gene expression and overall connectivity between GSE117613 and GSE34404 were positive, and significant *P*-values were obtained in both the malaria-infected and noninfected groups (Fig. 5A). The results suggest that the GSE117613 and GSE34404 datasets are comparable. In the control group, the green, brown, black, red, and green-yellow modules were identified as highly conserved between the GSE117613 and GSE34404 datasets because their values of “Zsummary. Pres” were more than 10 (Fig. 5B). Then, the genes in these conserved modules were subjected to GO function enrichment analysis (Additional file 4: Fig. S4). Genes in the brown, black, and green-yellow modules could not be clustered. In the malaria-infected group, the red, blue, brown, turquoise, yellow, and black modules were identified as highly conserved (Fig. 5C). Genes in the red and black modules could not be clustered. The GO enrichment results of each module are shown in a dot plot (Additional file 5: Fig. S5). The result shows that there are some similar biological activities between the noninfected group and the malaria-infected group, including translation initiation, ribosome synthesis, and ubiquitin-protein transferase activity. Importantly, many different biological activities were found in the malaria-infected group compared with the normal control group. Many genes in the malaria-infected group are significantly associated with neutrophil degranulation and activation, myeloid cell and erythrocyte differentiation, and homeostasis, which was not observed in the noninfected group (Fig. 6A ,B).

### Immunophenotyping of whole blood samples based on ssGSEA

**Fig. 7:**
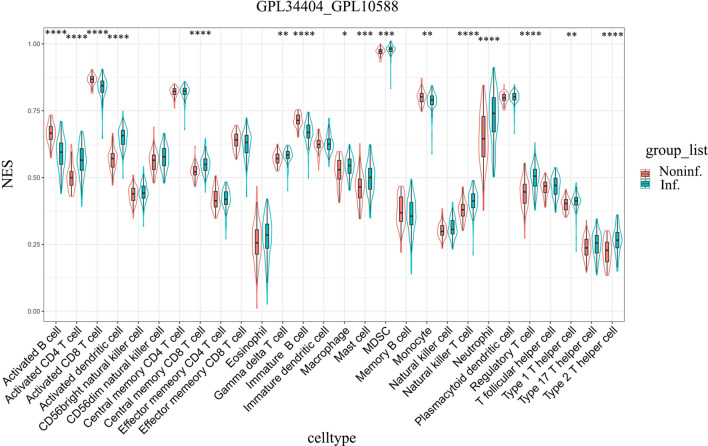
Immunophenotyping of whole-blood samples in malaria-infected and uninfected groups in GSE34404 datasets based on ssGSEA. The ssGSEA scores (enrichment level) were compared between noninfected and malaria-infected groups in each dataset. The statistical analyses were performed using the unpaired *t*-test. * p < 0.05, ** p < 0.01, *** p < 0.001, **** p < 0.0001

**Fig. 8:**
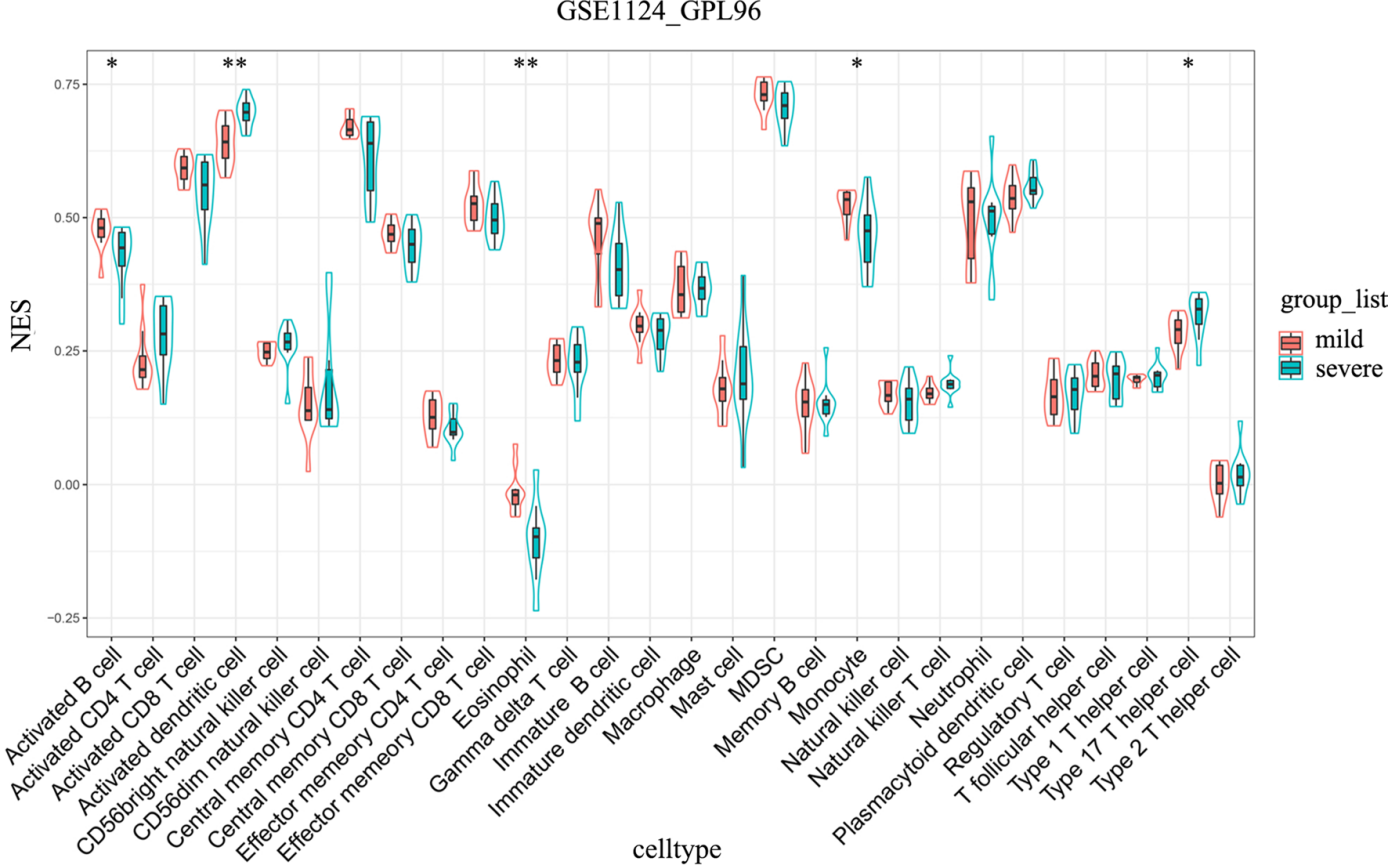
Immunophenotyping of whole-blood samples in the mild and severe malaria group in GSE1124 datasets based on ssGSEA. The ssGSEA scores (enrichment level) were compared between the mild malaria group and severe malaria group in each dataset. The statistical analyses were performed using the unpaired *t*-test. * p < 0.05, ** p < 0.01

The different immune cell types were also investigated in whole blood samples from the malaria-infected and noninfected groups. ssGSEA was used to assess and grade the enrichment of 28 immune signatures for each sample. Compared with those of the noninfected groups, the whole blood of the malaria-infected groups had significantly higher numbers of activated dendritic cells and type 2 T helper cells but significantly lower numbers of activated B cells, immature B cells, and monocytes in all three datasets (GSE1124-GPL96, GSE117613, and GSE34404) (Fig. 7, Additional file 6: Fig. S6 and Additional file 7: Fig. S7). Additionally, there were significantly higher umbers of neutrophils in the whole blood of the malaria-infected group than those of noninfected in both the GSE34404 and GSE1124-GPL96 datasets (Fig. 7 and Additional file 6: Fig. S6). GSE117613 dataset also had a higher number of neutrophils in the infected group than the noninfected group, but the differences were not statistically significant (Additional file 7: Fig. S7). Furthermore, ssGSEA was performed to investigate the different immune cell types in the whole blood of the mild and severe malaria groups in the GSE1124-GPL96 and GSE35858 datasets. Uncomplicated malaria was considered the mild malaria group, while complicated, severe malaria anaemia, and cerebral malaria was considered the severe malaria group. The results showed that compared with the mild malaria group, the severe malaria group had significantly higher numbers of activated dendritic cells and significantly lower numbers of monocytes in the GSE1124-GPL96 and GSE35858 datasets (Fig. 8 and Additional file 8: Fig. S8). It is worth noting that there were no differences in the number of neutrophils between the mild and severe malaria groups in these datasets. To confirm this result, the DEGs between mild malaria and severe malaria groups in the GSE1124-GPL96 and GSE35858 datasets were identified by the MetaIntegrator R package, and GO enrichment was performed. The results showed no neutrophil-associated functional enrichment (Additional file 9: Fig. S9).

## Discussion

In the present study, a systematic multicohort analysis approach was applied to comprehensively re-analyse publicly available microarray datasets to identify significant differentially regulated signatures in malaria-infected people relative to noninfected healthy individuals. Compared with the individual analyses of every single dataset, the multicohort analysis provided increased power in detecting the DEGs with high confidence, reducing the large number of false-positive markers detected in the individual study analyses.

Neutrophils are immune cells with unique biological features that provide potent antimicrobial properties [12, 13]. It has been shown that neutrophils have a dual role in malaria, contributing to both the pathogenesis and control of *Plasmodium* infections [14]. Changes in peripheral blood neutrophil levels have been described in *Plasmodium* spp. infections [15–17]. However, in naturally infected individuals, patterns of change in peripheral blood neutrophil numbers vary among the cohort studied [16–19]. Performing meta-analysis using three different systematic multicohort analysis methods, the result shows that neutrophil activation and neutrophil-mediated immunity were involved in the human responses to natural malaria infections. The data suggests that malaria-induced neutrophil activation and degranulation deserve further investigation. However, neutrophil cell enrichment and activation were not significantly different between mild and severe malaria groups. It has been reported that the neutrophil granule proteins MMP8, OLFM4, DEFA3, and ELANE are increased in severe malaria versus uncomplicated malaria [1]. Additionally, activated neutrophils are associated with iRBC cerebral sequestration in children with cerebral malaria (CM) [20]. Further studies are required to investigate the roles of neutrophils in malaria severity, particularly in CM.

Translational control of gene expression provides the cell with a rapid response to external triggers or other cues by changing its proteome without de novo mRNA synthesis. It has been reported that *Leishmania* spp*.* and *Toxoplasma gondii* can alter the host’s translation control and protein synthesis [21, 22]. Malaria parasites have also been reported to inhibit the host’s CXCL10 synthesis in monocytes by disrupting the association of host ribosomes with CXCL10 transcripts [23]. In the present study, the multicohort human whole blood transcriptional data showed that malaria infection can downregulate host genes in ribosome synthesis, protein translation, and protein localization and upregulate host cell division-related genes. Although the results of the multicohort WGCNA showed that there were conserved modules related to ribosome synthesis and protein translation in both the malaria-infected and noninfected groups, this analysis method cannot reflect the levels of change in ribosome synthesis- and protein translation-related genes in the two groups. Additionally, multicohort WGCHA analysis showed that malaria-infected groups had a conserved module related to erythrocyte differentiation and homeostasis, which was not shown in the control group. These results indicate that malaria parasites may facilitate their survival in human blood by inhibiting the synthesis of host immune-related proteins and promoting the division of red blood cells.

In this study, ssGSEA of the immune cell profile of peripheral blood was also performed in each sample. The results showed that activation of dendritic cells and type 2 T helper cells as well as inhibition of B cells, immature B cells, monocytes in the malaria-infected group relative to the noninfected group. It has been well established that B cells and antibodies are critical for controlling *Plasmodium* infection and for providing immunity to reinfection [24–27]. In recent years, a series of field studies have suggested that B memory cell function is impaired by *P. falciparum* infection [28–30]. The present study also found that *P. falciparum* infection can inhibit B cell activation and proliferation. It has been reported that *Plasmodium* DNA, haemozoin, or extracellular vesicles can impair the function of monocytes, and the monocyte count in the blood increases in primary *P. falciparum* infection [31]. However, at the transcriptional level, decreased monocyte-related gene enrichment was found in the *P. falciparum*-infected group compared to the noninfected control group, suggesting inhibition of monocyte functions by the parasites.

It has long been suggested that an excessive host immune response may contribute to severe malaria in humans [32, 33]. In the present study, significantly higher enrichment of activated dendritic cells and significantly lower enrichment of monocytes were found in the peripheral blood of the severe malaria group than in the mild malaria group. It has been reported that dendritic cells can induce immune-mediated pathology, including a life-threatening syndrome of cerebral malaria in murine studies [34, 35]. These observations suggest that increased dendritic cell activation may be related to severe malaria in *Plasmodium falciparum* infection. Monocytes are reported to be a key component in controlling parasite burden and protecting the host from malaria disease. Additionally, they play important roles in the pathogenesis of cerebral malaria [30]. It has been reported that the monocyte to neutrophil ratio is associated with severe malaria, especially in semi-immune patients [16]. Low monocyte to neutrophil ratio may indicate a risk of developing complicated malaria [36]. The findings in the present study are consistent with those reports. This multicohort study found that monocyte-related gene enrichment decreased in the severe malaria group relative to the mild malaria group, while neutrophils were not changed significantly between severe malaria and mild malaria groups. The role of monocytes in severe malaria requires further investigation.

## Conclusions

This study, using data from three different systematic multicohort analyses, identifies highly robust human response signatures in *Plasmodium falciparum* infection and disease progression. These results extend the understanding of the host immune response against malaria infections and may provide a basis for developing disease control measures including protective malaria vaccines.

## Supplementary Information

**Additional file 1: FigureS1. ** The receiver operating characteristic (ROC) curves of DEG expression in differentiating the malaria-infected group from the noninfected group in discovery cohorts GSE117613, GSE1124-GPL96, and GSE34404 and validation cohort GSE35858.

**Additional file 2: Figure S2. ** Genes in M2 modules. M2 module of DEGs was identified using Molecular Complex Detection (MCODE) plug-in in Cytoscape. Genes in red indicate upregulated genes, while genes in blue indicate downregulated genes in the malaria-infected group compared to the noninfected group.

**Additional file 3: FigureS3. ** Functional enrichment of upregulated genes and downregulated genes in the M1 module. A, GO enrichment analysis of upregulated genes. B, GO enrichment analysis of downregulated genes. 'Gene ratio' is the percentage of total DEGs in the given GO term. The size of the dots represents the number of genes in DEGs associated with the GO term and the colour of the dots represents the *P*-adjusted values.

**Additional file 4: Figure.S4. ** GO enrichment of highly conserved modules between noninfected groups of GSE117613 and GSE34404 datasets analysed by multicohort WGCNA. A, GO enrichment of red module. B, GO enrichment of green module. 'Gene ratio' is the percentage of total DEGs in the given GO term. The size of the dots represents the number of genes in DEGs associated with the GO term and the colour of the dots represents the *P*-adjusted values.

**Additional file 5: FigureS5. ** GO enrichment of highly conserved modules between malaria-infected groups of GSE117613 and GSE34404 datasets analysed using multicohort WGCNA. A, GO enrichment of blue module. B, GO enrichment of turquoise module. C, GO enrichment of brown module. D, GO enrichment of yellow module. 'Gene ratio' is the percentage of total DEGs in the given GO term. The size of the dots represents the number of genes in DEGs associated with the GO term and the colour of the dots represents the *P*-adjusted values.

**Additional file 6: FigureS6. ** Immunophenotyping of whole-blood samples in malaria-infected and uninfected groups in GSE1124 datasets based on ssGSEA. The ssGSEA scores (enrichment level) were compared between noninfected and malaria-infected groups in each dataset. The statistical analyses were performed using the unpaired *t*-test. * p<0.05, ** p<0.01, *** p<0.001, **** p<0.0001.

**Additional file 7:** **Figure** **S7. ** Immunophenotyping of whole-blood samples in malaria-infected and uninfected groups in GSE117613 datasets based on ssGSEA. The ssGSEA scores (enrichment level) were compared between noninfected and malaria-infected groups in each dataset. The statistical analyses were performed using the unpaired *t*-test. * p<0.05, ** p<0.01, *** p<0.001, **** p<0.0001.

**Additional file 8: Figure S8. ** Immunophenotyping of whole-blood samples in the mild and severe malaria group in GSE35858 datasets based on ssGSEA. The ssGSEA scores (enrichment level) were compared between the mild malaria group and severe malaria group in each dataset. The statistical analyses were performed using the unpaired *t*-test. * p<0.05, ** p<0.01, *** p<0.001.

**Additional file 9: FigureS9.** GO enrichment analysis of differentially expressed genes between mild malaria and severe malaria in GSE1124-GPL96 and GSE35858 datasets.

**Additional file 10: Table S1.** The subsets of genes and immune cell types identified from the ImmPort database.

**Additional file 11: Table S2.** The upregulated genes in malaria-infected relative to noninfected individuals in the discovery datasets.

**Additional file 12: Table S3.** The downregulated genes in malaria-infected relative to noninfected individuals in the discovery datasets.

## Data Availability

The datasets used and/or analysed during the current study are available from the corresponding author upon request.

## Abbreviations

DEGs: Differentially expressed genes
CHMI: Controlled human malaria infection
IFN-β: Interferon-beta
PPI: Protein–protein interaction
WGCNA: Weighted gene coexpression network analysis
ssGSEA: Single-sample gene set enrichment analysis
GEO: Gene Expression Omnibus
PBMCs: Peripheral blood mononuclear cells
ROC: Receiver operating characteristic curves
GO: Gene Ontology
BP: Biological process
CC: Cellular component
MF: Molecular function
STRING: The Retrieval of Interacting Genes database
MCODE: The molecular complex detection
AUC: The area under the receiver operating characteristic curve values
CM: Cerebral malaria
