# Supplementary figures and images for: Multicohort transcriptome analysis of whole blood identifies robust human response signatures in Plasmodium falciparum infections
Source: Malar J. 2022 Nov 15;21:333. doi: 10.1186/s12936-022-04374-5 (PMC9664782; doi:10.1186/s12936-022-04374-5)

A

multipleROC

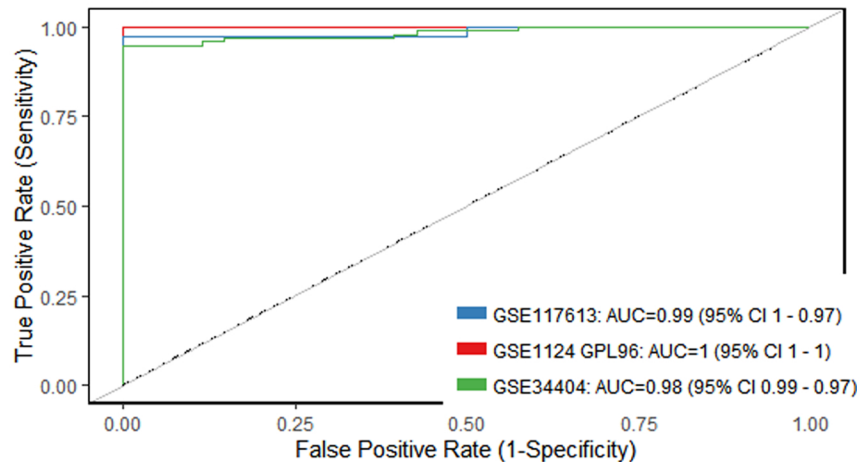

B

GSE35858

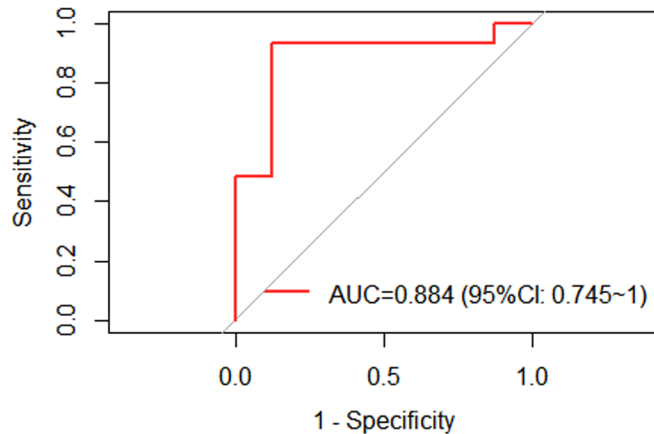

Supplement: Supplementary file 1 — Additional file 1: FigureS1. The receiver operating characteristic (ROC) curves of DEG expression in differentiating the malaria-infected group from the noninfected group in discovery cohorts GSE117613, GSE1124-GPL96, and GSE34404 and validation cohort GSE35858. [file 12936_2022_4374_MOESM1_ESM.pdf]

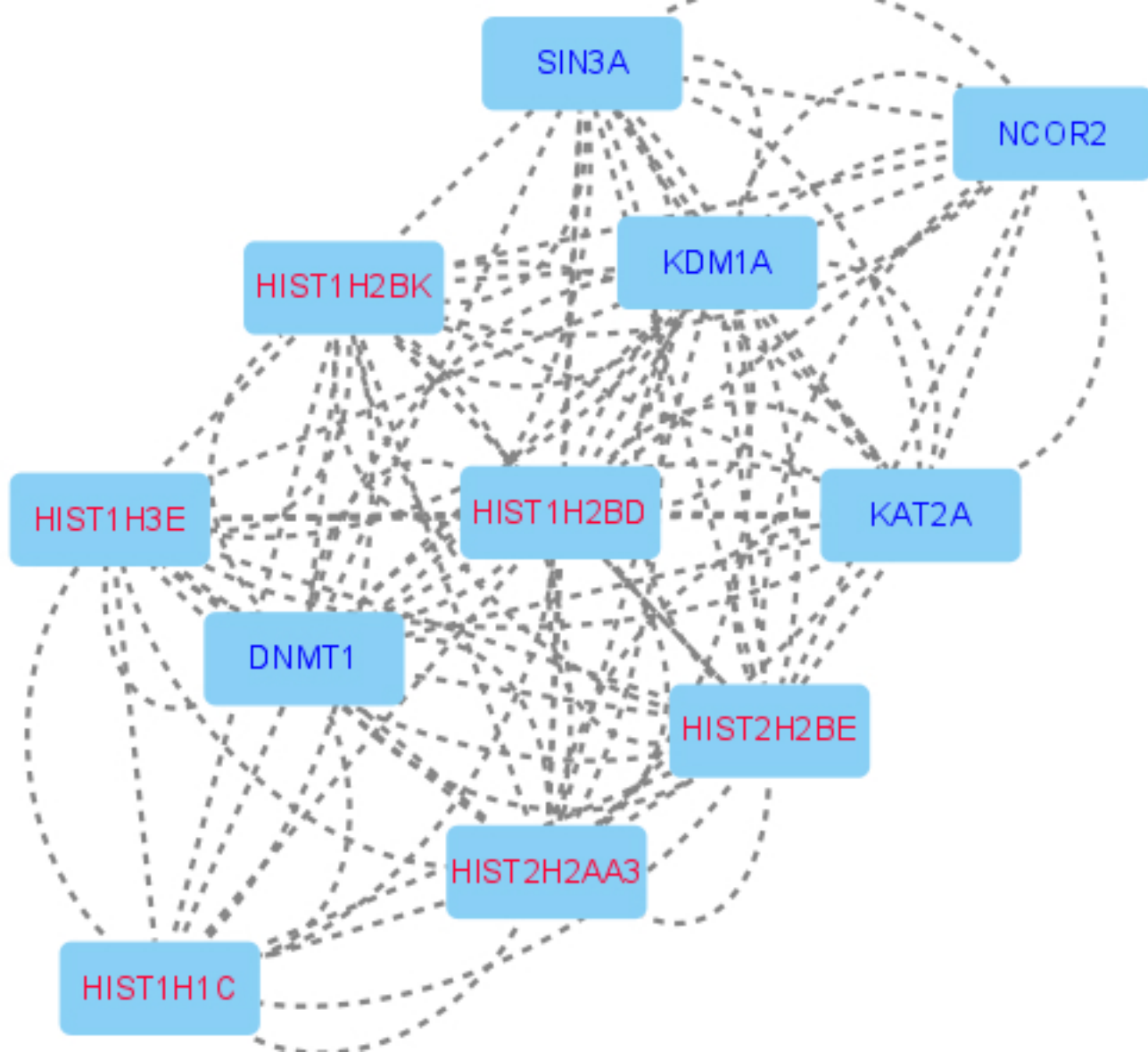

Supplement: Supplementary file 2 — Additional file 2: Figure S2. Genes in M2 modules. M2 module of DEGs was identified using Molecular Complex Detection (MCODE) plug-in in Cytoscape. Genes in red indicate upregulated genes, while genes in blue indicate downregulated genes in the malaria-infected group compared to the noninfected group. [file 12936_2022_4374_MOESM2_ESM.pdf]

A

## GO enrichment of red module

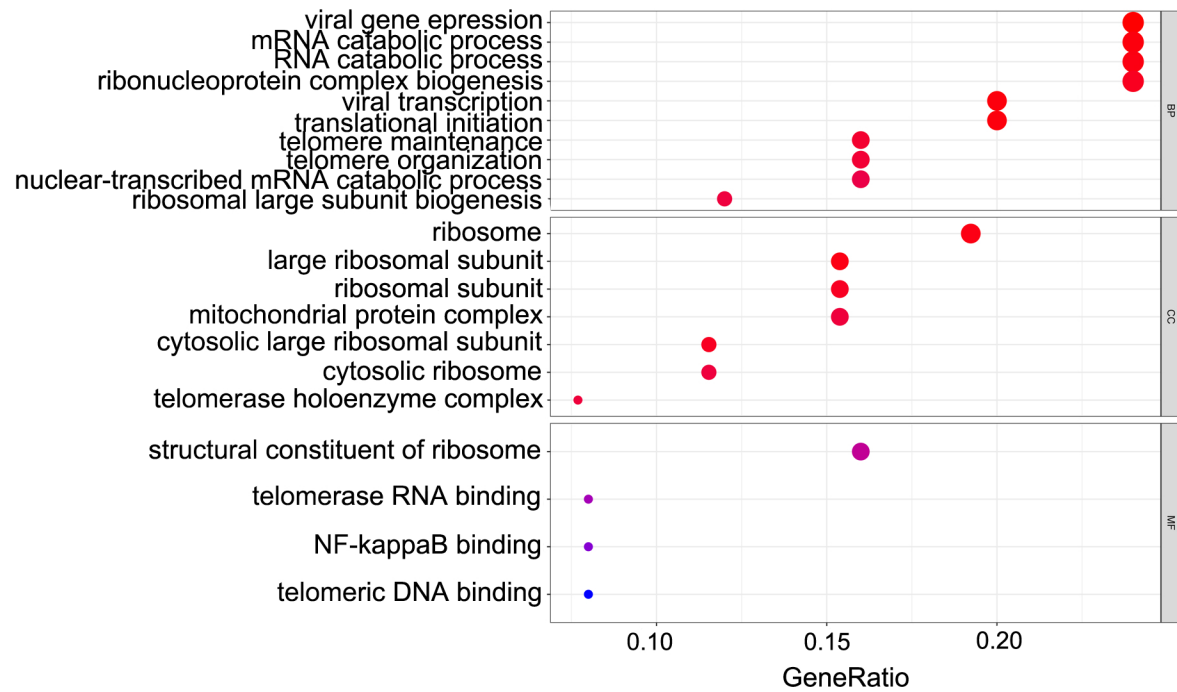

B

## GO enrichment of green module

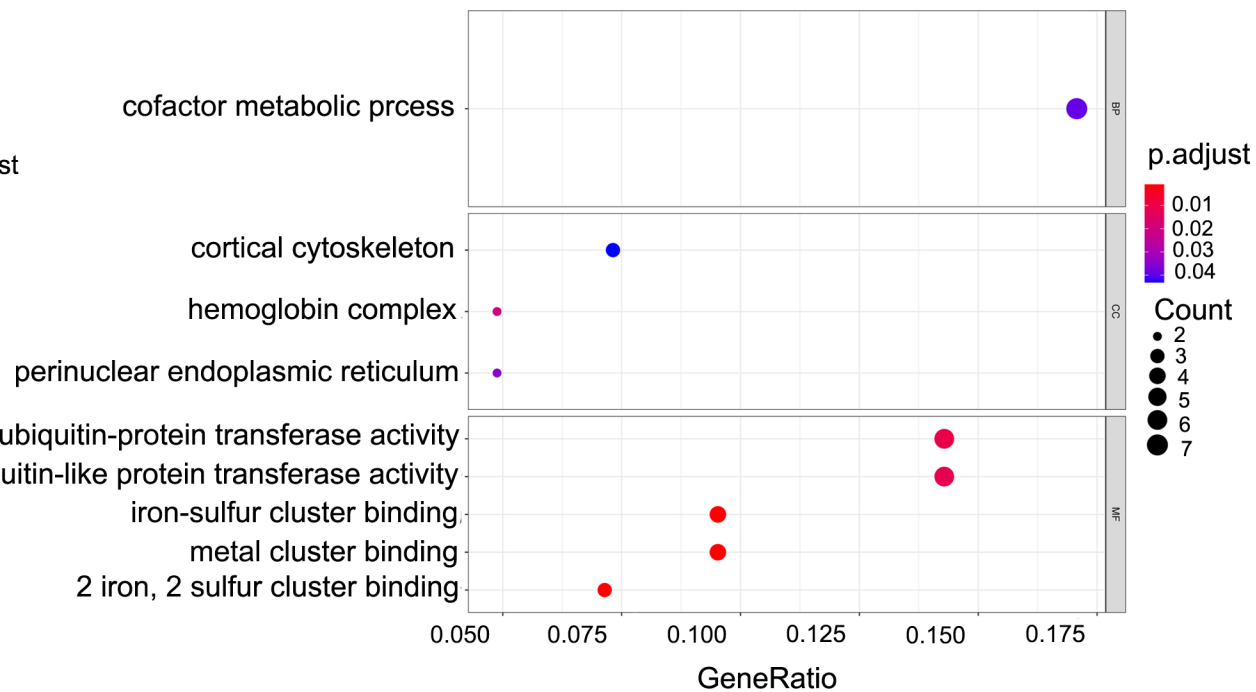

Supplement: Supplementary file 4 — Additional file 4: Figure.S4. GO enrichment of highly conserved modules between noninfected groups of GSE117613 and GSE34404 datasets analysed by multicohort WGCNA. A, GO enrichment of red module. B, GO enrichment of green module. 'Gene ratio' is the percentage of total DEGs in the given GO term. The size of the dots represents the number of genes in DEGs associated with the GO term and the colour of the dots represents the P-adjusted values. [file 12936_2022_4374_MOESM4_ESM.pdf]

# GSE1124\_GPL96

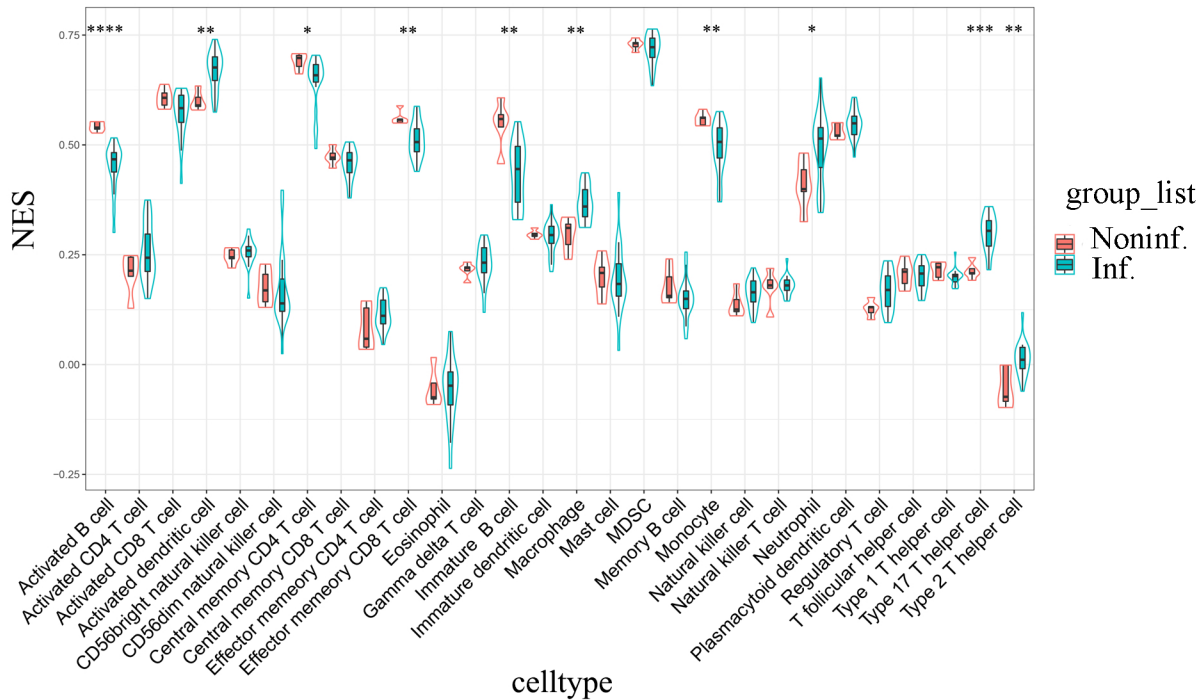

Supplement: Supplementary file 6 — Additional file 6: FigureS6. Immunophenotyping of whole-blood samples in malaria-infected and uninfected groups in GSE1124 datasets based on ssGSEA. The ssGSEA scores (enrichment level) were compared between noninfected and malaria-infected groups in each dataset. The statistical analyses were performed using the unpaired t-test. * p<0.05, ** p<0.01, *** p<0.001, **** p<0.0001. [file 12936_2022_4374_MOESM6_ESM.pdf]

# GSE117613\_GPL10588

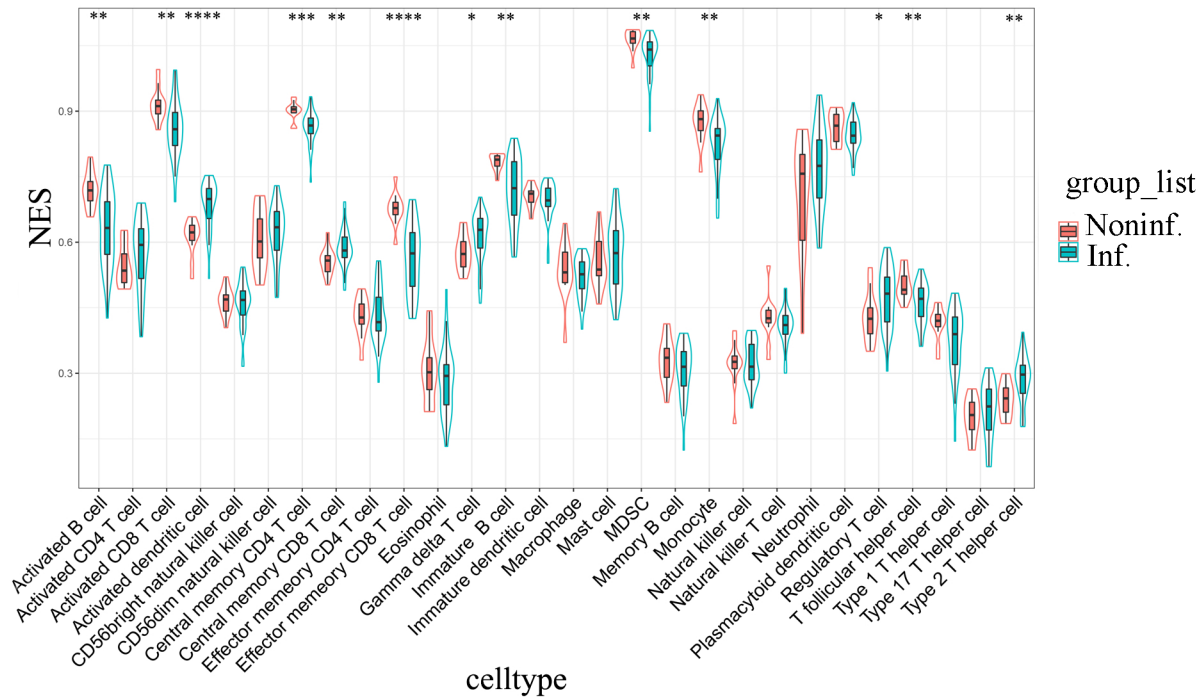

Supplement: Supplementary file 7 — Additional file 7: Figure S7. Immunophenotyping of whole-blood samples in malaria-infected and uninfected groups in GSE117613 datasets based on ssGSEA. The ssGSEA scores (enrichment level) were compared between noninfected and malaria-infected groups in each dataset. The statistical analyses were performed using the unpaired t-test. * p<0.05, ** p<0.01, *** p<0.001, **** p<0.0001. [file 12936_2022_4374_MOESM7_ESM.pdf]

# GSE35858\_GPL15240

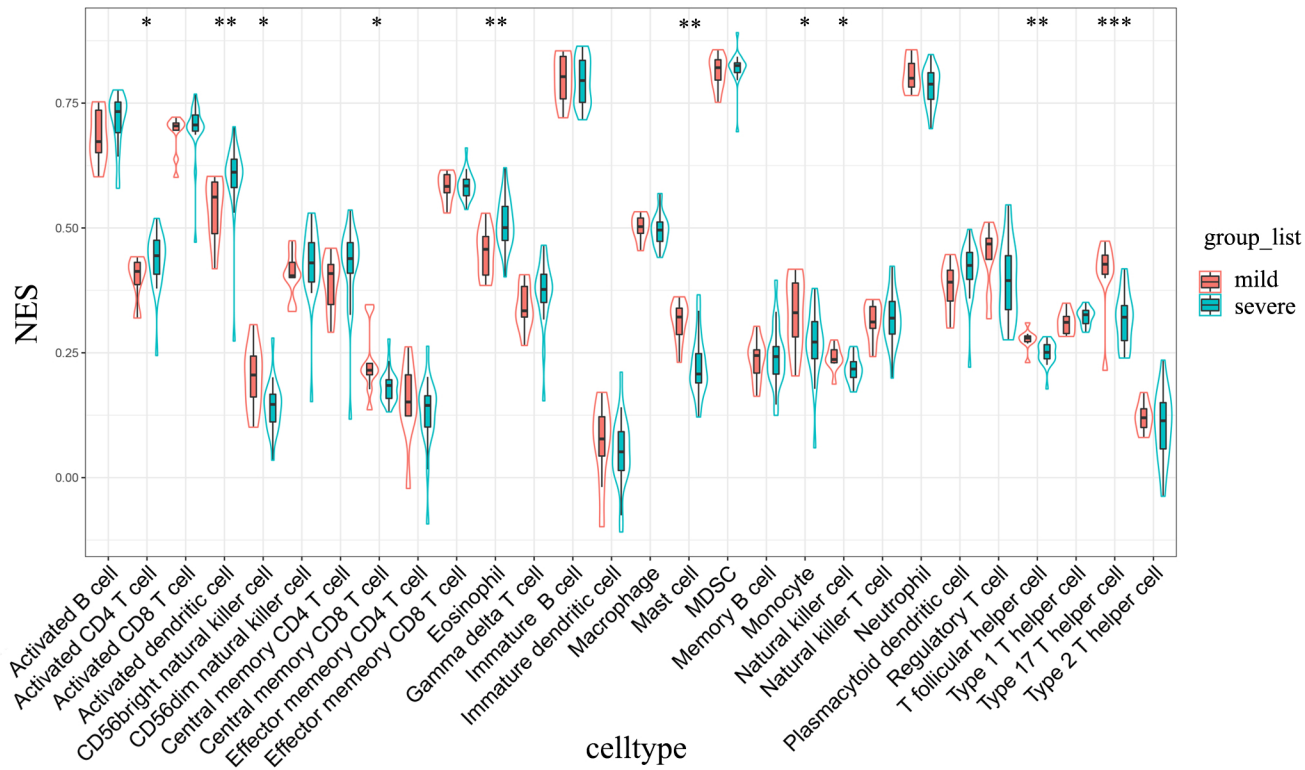

Supplement: Supplementary file 8 — Additional file 8: Figure S8. Immunophenotyping of whole-blood samples in the mild and severe malaria group in GSE35858 datasets based on ssGSEA. The ssGSEA scores (enrichment level) were compared between the mild malaria group and severe malaria group in each dataset. The statistical analyses were performed using the unpaired t-test. * p<0.05, ** p<0.01, *** p<0.001. [file 12936_2022_4374_MOESM8_ESM.pdf]

anaphase-promoting complex-dependet catabolic process

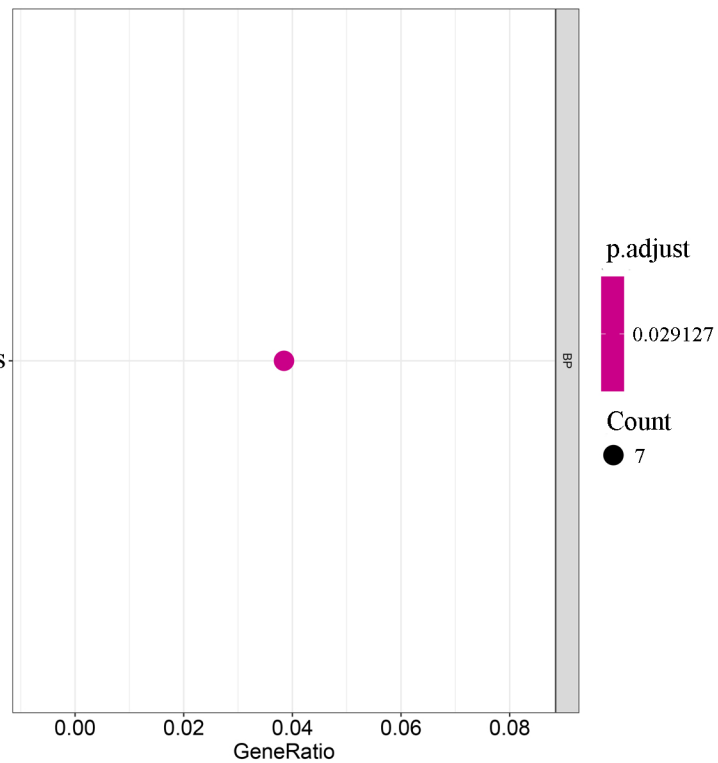

Supplement: Supplementary file 9 — Additional file 9: FigureS9. GO enrichment analysis of differentially expressed genes between mild malaria and severe malaria in GSE1124-GPL96 and GSE35858 datasets. [file 12936_2022_4374_MOESM9_ESM.pdf]
