# Supplementary material for: Multicohort transcriptome analysis of whole blood identifies robust human response signatures in Plasmodium falciparum infections
Source: Malar J. 2022 Nov 15;21:333. doi: 10.1186/s12936-022-04374-5 (PMC9664782; doi:10.1186/s12936-022-04374-5)
Supplement: Supplementary file 3 — Additional file 3: FigureS3. Functional enrichment of upregulated genes and downregulated genes in the M1 module. A, GO enrichment analysis of upregulated genes. B, GO enrichment analysis of downregulated genes. 'Gene ratio' is the percentage of total DEGs in the given GO term. The size of the dots represents the number of genes in DEGs associated with the GO term and the colour of the dots represents the P-adjusted values. [file 12936_2022_4374_MOESM3_ESM.pdf]

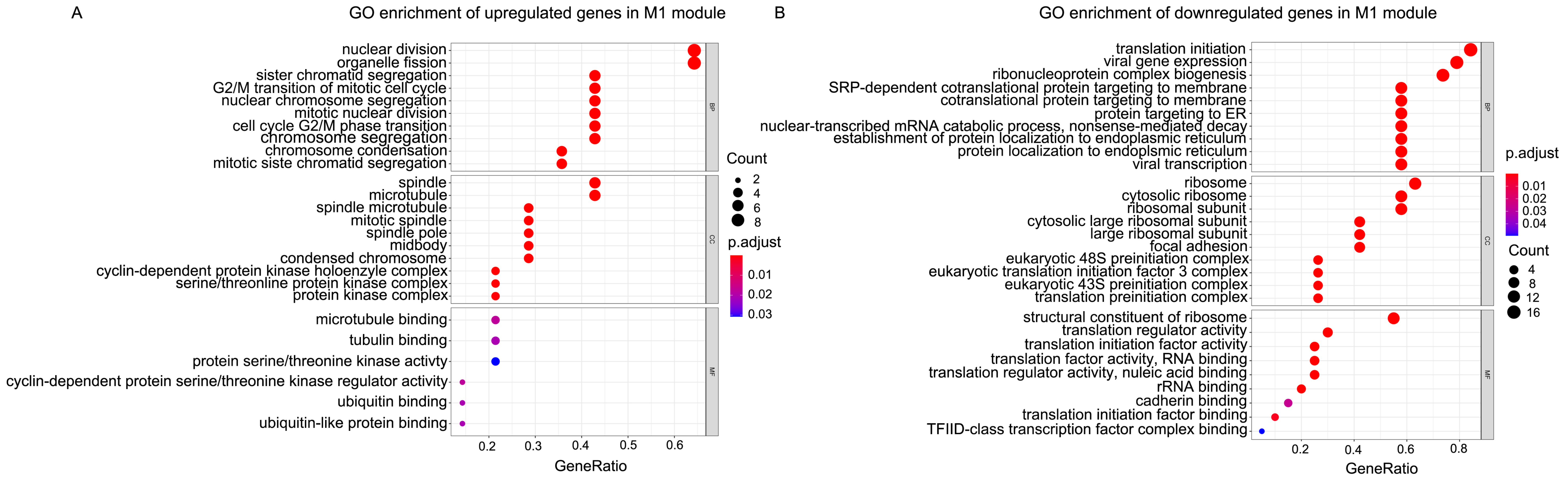

A
GO enrichment of upregulated genes in M1 module
nuclear division
organelle fission
sister chromatid segregation
G2/M transition of mitotic cell cycle
nuclear chromosome segregation
mitotic nuclear division
cell cycle G2/M phase transition
chromosome segregation
chromosome condensation
mitotic siste chromatid segregation
spindle
microtubule
spindle microtubule
mitotic spindle
spindle pole
midbody
condensed chromosome
cyclin-dependent protein kinase holoenzyle complex
serine/threonline protein kinase complex
protein kinase complex
microtubule binding
tubulin binding
protein serine/threonine kinase activty
cyclin-dependent protein serine/threonine kinase regulator activity
ubiquitin binding
ubiquitin-like protein binding
BP
CC
MF
0.2
0.3
0.4
0.5
0.6
GeneRatio
Count
2
4
6
8
p.adjust
0.01
0.02
0.03
B
GO enrichment of downregulated genes in M1 module
translation initiation
viral gene expression
ribonucleoprotein complex biogenesis
SRP-dependent cotranslational protein targeting to membrane
cotranslational protein targeting to membrane
protein targeting to ER
nuclear-transcribed mRNA catabolic process, nonsense-mediated decay
establishment of protein localization to endoplasmic reticulum
protein localization to endoplsmic reticulum
viral transcription
ribosome
cytosolic ribosome
ribosomal subunit
cytosolic large ribosomal subunit
large ribosomal subunit
focal adhesion
eukaryotic 48S preinitiation complex
eukaryotic translation initiation factor 3 complex
eukaryotic 43S preinitiation complex
translation preinitiation complex
structural constituent of ribosome
translation regulator activity
translation initiation factor activity
translation factor activity, RNA binding
translation regulator activity, nuleic acid binding
rRNA binding
cadherin binding
translation initiation factor binding
TFIID-class transcription factor complex binding
BP
CC
MF
0.2
0.4
0.6
0.8
GeneRatio
p.adjust
0.01
0.02
0.03
0.04
Count
4
8
12
16
