# Supplementary material for: Multicohort transcriptome analysis of whole blood identifies robust human response signatures in Plasmodium falciparum infections
Source: Malar J. 2022 Nov 15;21:333. doi: 10.1186/s12936-022-04374-5 (PMC9664782; doi:10.1186/s12936-022-04374-5)
Supplement: Supplementary file 5 — Additional file 5: FigureS5. GO enrichment of highly conserved modules between malaria-infected groups of GSE117613 and GSE34404 datasets analysed using multicohort WGCNA. A, GO enrichment of blue module. B, GO enrichment of turquoise module. C, GO enrichment of brown module. D, GO enrichment of yellow module. 'Gene ratio' is the percentage of total DEGs in the given GO term. The size of the dots represents the number of genes in DEGs associated with the GO term and the colour of the dots represents the P-adjusted values. [file 12936_2022_4374_MOESM5_ESM.pdf]

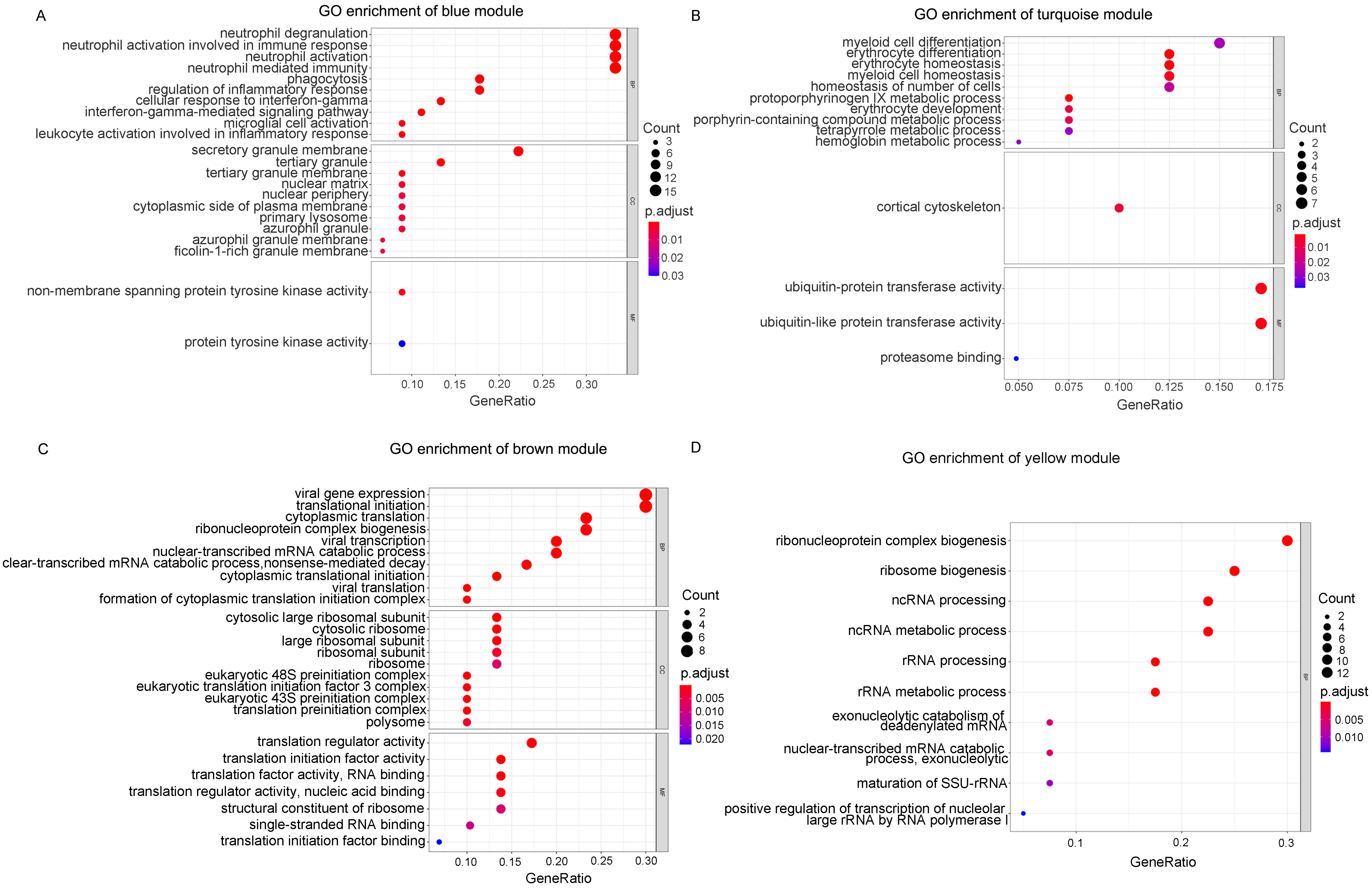

A
GO enrichment of blue module
neutrophil degranulation
neutrophil activation involved in immune response
neutrophil activation
neutrophil mediated immunity
phagocytosis
regulation of inflammatory response
cellular response to interferon-gamma
interferon-gamma-mediated signaling pathway
microglial cell activation
leukocyte activation involved in inflammatory response
secretory granule membrane
tertiary granule
tertiary granule membrane
nuclear matrix
nuclear periphery
cytoplasmic side of plasma membrane
primary lysosome
azurophil granule
azurophil granule membrane
ficolin-1-rich granule membrane
non-membrane spanning protein tyrosine kinase activity
protein tyrosine kinase activity
BP
CC
MF
0.10 0.15 0.20 0.25 0.30
GeneRatio
Count
3
6
9
12
15
p.adjust
0.01
0.02
0.03
B
GO enrichment of turquoise module
myeloid cell differentiation
erythrocyte differentiation
erythrocyte homeostasis
myeloid cell homeostasis
homeostasis of number of cells
protoporphyrinogen IX metabolic process
erythrocyte development
porphyrin-containing compound metabolic process
tetrapyrrole metabolic process
hemoglobin metabolic process
cortical cytoskeleton
ubiquitin-protein transferase activity
ubiquitin-like protein transferase activity
proteasome binding
BP
CC
MF
0.050 0.075 0.100 0.125 0.150 0.175
GeneRatio
Count
2
3
4
5
6
7
p.adjust
0.01
0.02
0.03
C
GO enrichment of brown module
viral gene expression
translational initiation
cytoplasmic translation
ribonucleoprotein complex biogenesis
viral transcription
nuclear-transcribed mRNA catabolic process
clear-transcribed mRNA catabolic process,nonsense-mediated decay
cytoplasmic translational initiation
viral translation
formation of cytoplasmic translation initiation complex
cytosolic large ribosomal subunit
cytosolic ribosome
large ribosomal subunit
ribosomal subunit
ribosome
eukaryotic 48S preinitiation complex
eukaryotic translation initiation factor 3 complex
eukaryotic 43S preinitiation complex
translation preinitiation complex
polysome
translation regulator activity
translation initiation factor activity
translation factor activity, RNA binding
translation regulator activity, nucleic acid binding
structural constituent of ribosome
single-stranded RNA binding
translation initiation factor binding
BP
CC
MF
0.10 0.15 0.20 0.25 0.30
GeneRatio
Count
2
4
6
8
p.adjust
0.005
0.010
0.015
0.020
D
GO enrichment of yellow module
ribonucleoprotein complex biogenesis
ribosome biogenesis
ncRNA processing
ncRNA metabolic process
rRNA processing
rRNA metabolic process
exonucleolytic catabolism of deadenylated mRNA
nuclear-transcribed mRNA catabolic process, exonucleolytic
maturation of SSU-rRNA
positive regulation of transcription of nucleolar large rRNA by RNA polymerase I
BP
0.1 0.2 0.3
GeneRatio
Count
2
4
6
8
10
12
p.adjust
0.005
0.010
